# Mepitel Film Versus StrataXRT in Managing Radiation Dermatitis in an Intra‐Patient Controlled Clinical Trial of 80 Postmastectomy Patients

**DOI:** 10.1111/1754-9485.13850

**Published:** 2025-04-14

**Authors:** Patries Herst, Mary van Schalkwyk, Natasha Baker, Rebecca Thyne, Kelly Dunne, Kylie Moore, Freya Jackson, Kendal Beaven, Lucy Rutten, Gemma McKee, Robin Willink, Melissa James

**Affiliations:** ^1^ Department of Radiation Therapy University of Otago Wellington New Zealand; ^2^ Canterbury Regional Cancer and Haematology Service, Christchurch Hospital Christchurch New Zealand; ^3^ Regional Cancer Treatment Service, Palmerston North Hospital Palmerston North New Zealand; ^4^ Wellington Blood and Cancer Centre, Wellington Hospital Wellington New Zealand; ^5^ Biostatistics Group University of Otago Wellington New Zealand; ^6^ Department of Medicine, Christchurch Hospital University of Otago Christchurch Christchurch New Zealand

**Keywords:** breast cancer, mastectomy, Mepitel film, moist desquamation, radiation therapy, skin reactions, StrataXRT

## Abstract

**Background:**

Mepitel film decreases the severity of radiation dermatitis in breast cancer patients, but its application is resource‐intensive. Many departments therefore use StrataXRT, a gel that patients apply themselves. We compared the protective effects of Mepitel film and StrataXRT on radiation dermatitis during and immediately after radiation therapy.

**Methods:**

This phase III multicentre randomised intra‐patient‐controlled clinical trial recruited breast cancer patients receiving radiation therapy following mastectomy in three New Zealand Hospitals. Lateral and medial aspects of the chest wall were randomised to either Mepitel film or StrataXRT. Overall skin reaction severity was measured using RISRAS and RTOG once a week during treatment and for 4 weeks after treatment completion. The primary outcome was moist desquamation (with a non‐inferiority margin of 7.5%); secondary outcomes were overall skin reaction severity, patient tolerability, satisfaction and cost.

**Results:**

Between June 2021 and May 2024, 93 patients were recruited, and 80 patients completed the trial. The absolute difference in moist desquamation rates was 6% lower under Mepitel film (*p* = 0.413, 95% CI ‐5%, 18%). Overall skin reaction severity was significantly lower for Mepitel film (Researcher RISRAS: *p* = 0.022; RTOG: *p* = 0.011). Mepitel film was cheaper to apply but was less well tolerated, with poor skin adherence being an issue for many patients.

**Conclusion:**

The study did not show non‐inferiority for StrataXRT; overall skin reaction severity and costs were significantly lower under Mepitel film; however, StrataXRT was better tolerated.

**Trial Registration:** ACTRN12621000124831

## 1 Introduction

Breast cancer is the most common cancer in females, with approximately 3500 women diagnosed in New Zealand each year (https://teaho.govt.nz/cancer‐numbers/cancer‐rates/breast‐cancer‐rate). Radiation therapy is part of the cancer journey of more than half of all breast cancer patients. Radiation treatment is often indicated after a mastectomy for breast cancer to decrease the risk of recurrence [1, 2]. The most common acute side effect of radiation therapy is radiation dermatitis, characterised by erythema and, in more severe cases, loss of skin integrity leading to moist desquamation. Patients describe radiation dermatitis as painful, burning and/or itchy and affecting their quality of life [3]. Although acute symptoms usually self‐resolve within months after completion of treatment, severe radiation dermatitis can compromise treatment adherence and thus patient survival as well as quality of life. Long‐term sequelae include fibrosis, hyperpigmentation and telangiectasia, which further affect patient morbidity and quality of life [3]. Preventing severe acute radiation dermatitis is therefore important for patients whose skin receives a high dose of radiation, such as breast [4, 5] head and neck [6, 7] and pelvic cancers [8].

For several decades, many topical interventions to better manage acute radiation dermatitis have been investigated, with systematic reviews every couple of years unable to recommend any intervention. In 2023, the Multinational Association of Supportive Care in Cancer (MASCC) Oncodermatology Study Group published a critical review of 149 RCTs in Lancet Oncology [9, 10]. This study used a modified Delphi consensus process with recommendations from an international 42‐member specialist panel using a 75% consensus threshold. Mepitel film was recommended for use in breast cancer patients (76%), with a near consensus threshold (74%) in head and neck cancer patients [10]. Mepitel film is a Safetac‐based soft silicone dressing that adheres closely to the skin to create a physical barrier, preventing friction damage to the irradiated skin, allowing the skin to heal in between fractions, whilst minimising inflammation and providing a moist wound environment conducive to healing [11]. Mepitel film has been shown to decrease the number of patients developing severe skin reactions in nine breast cancer studies, including three randomised controlled clinical trials (RCTs) [4, 12, 13], recently reviewed in [14, 15]. Two of three RCTs in head and neck cancer patients also found Mepitel film is superior when compared with aqueous cream and biafine cream [6, 7]. A third head and neck RCT that closed early due to a lack of efficacy and poor tolerance used mometasone furoate as standard care [16], another intervention recommended by MASCC [10].

Widespread use of Mepitel film has remained limited because application by a health care professional is required, straining departmental resources. This has resulted in clinicians using a silicone‐based gel, StrataXRT, which is applied by patients themselves and forms a thin film on the skin upon drying. Two RCTs have been published using StrataXRT in breast cancer patients [17, 18] and one in head and neck cancer patients [19], recently reviewed in [20]. Because of the limited clinical evidence currently available, StrataXRT was not recommended by MASCC [10]. Here, we directly compared the protective effects of Mepitel film and StrataXRT on radiation dermatitis in mastectomy patients undergoing radiation therapy in an intra‐patient‐controlled RCT.

## 2 Methodology

This randomised, intra‐patient controlled, multicentre clinical trial was conducted in Christchurch, Palmerstone North, and Wellington public hospitals in New Zealand between June 2021 and April 2024. Ethical approval was obtained from the University of Otago Ethics Committee (Health) in November 2020 (H20/130) and from the Northern B Health and Disability Ethics Committee in April 2021 (21/NTB/78); the trial is registered with the Australia New Zealand Clinical Trials Registry (ACTRN12621000124831). All participants gave written informed consent before the start of radiation therapy.

### 2.1 Trial Objectives

The primary objective was to study the difference in moist desquamation rates between Mepitel film‐covered skin patches and StrataXRT‐covered skin patches. Secondary objectives were to study differences in overall skin reaction severity, patient tolerability, satisfaction and costs.

### 2.2 Participants

All women and men receiving radiation therapy for breast cancer, following mastectomy, were screened for eligibility between June 2021 and April 2024. Specific exclusion criteria were previous radiation therapy to the chest wall, metastatic disease, breast reconstruction, a Karnofsky performance status score of less than 70, and those not being able to commit to the 4 weekly follow‐up visits.

### 2.3 Randomisation and Blinding

At the start of radiation treatment, the chest wall of each patient was divided into medial and lateral halves for randomisation to either Mepitel film or StrataXRT. Randomisation was based on computer‐generated randomisation charts. Because Mepitel film was applied by the research radiation therapists and StrataXRT was applied by the patients themselves, blinding was not possible.

### 2.4 Radiation Therapy

Patients were treated supine with their arms supported above their heads. Radiation therapy to the chest wall included both 3D conformal techniques (field in field) and inverse planning techniques (IMRT and VMAT) with some patients having a thin bolus (< 0.5 cm) applied over the scar/chest wall. Fractionation regimens included 26Gy in 5 fractions, 40.05Gy in 15 fractions and 50Gy in 25 fractions. Details of radiation therapy are shown in Table 2.

### 2.5 Application of Mepitel Film and StrataXRT

Our intra‐patient‐controlled approach allowed participants to experience both interventions and eliminated potential confounding patient‐ and treatment‐related factors. Both interventions were applied from the start of radiation treatment on the entire lateral or medial aspects of the chest wall and continued for 4 weeks after treatment completion. Mepitel film was applied by radiation therapists with the patient in the treatment position. Care was taken not to stretch the film with a 1 cm overlap between sheets as a single sheet has a negligible bolus effect [4]. Small pieces of curled film were trimmed with scissors; sheets were replaced when necessary. Mepitel film was generously donated by Molnlycke Healthcare LTD (Sweden). StrataXRT was applied by patients themselves twice a day. Patients were instructed to use a very small volume of StrataXRT, following Stratpharma's instructions, and wait until the gel had dried into a thin film (5–6 min) before covering the skin with clothing. StrataXRT was generously donated by Stratpharma LTD (Switzerland). Neither company has had any input in trial design or analysis and reporting of the results.

### 2.6 Skin Reaction Severity Measures

Skin reaction severity was quantified once a week during treatment and weekly for four weeks after completion of treatment. We used both the Radiation Induced Skin Reaction Assessment Scale (RISRAS) and the expanded Radiation Therapy Oncology Group (RTOG) scale [4]. The overall average RISRAS score was the sum of a researcher component, where the radiation therapist scores the visible extent of the skin reactions (erythema, dry and moist desquamation) and a patient component, where the patient scores the level of pain, itchiness and burning as well as the effect on daily life [4]. The RTOG grade was assigned by the radiation therapist as follows: 0—no change; 1—follicular faint or dull erythema; IIA—tender or bright erythema; IIB patchy—moist desquamation; III—confluent moist desquamation other than in skinfolds. Moist desquamation was deemed to be present if the researcher RISRAS score for moist desquamation was more than 0 or the maximum RTOG grade was IIB or III. All radiation therapists completed a workshop on scoring skin reaction severity using RISRAS and RTOG to minimise scorer variability.

### 2.7 Patient Satisfaction Measures

All patients were given a written exit questionnaire and were provided with a paid self‐addressed envelope to assist with compliance. The survey included questions about their experience in taking part in the trial and using Mepitel film and StrataXRT. The number of participants returning the questionnaire was recorded, as were their yes/no responses. Answers to open‐ended questions were grouped per theme using content analysis and are tabulated in Table 3.

### 2.8 Statistical Analysis

Our analysis was a (one‐sided) test of the null hypothesis that *“*the probability of moist desquamation under StrataXRT is greater than or equal to the probability of moist desquamation (MD) under Mepitel film plus a non‐inferiority margin of 7.5%*”*. This was carried out by calculating a 95% confidence interval for the difference in probabilities using the method of Tango [21] to examine whether the upper limit of this interval sat below the non‐inferiority margin of 7.5%.

For each of the three RISRAS analyses, we conducted a (two‐sided) 0.05‐level Wilcoxon signed‐rank test of the hypothesis “the distribution of RISRAS scores is the same for gel and film*”*. As this is a non‐parametric test, no parameter estimates are reported.

The RTOG data are on an ordinal scale with a small number of categories (grades). The null hypothesis was that “the probability that the grade under film exceeds the grade under gel is equal to the probability that the grade under gel exceeds the grade under film*”*. The corresponding (two‐sided) 0.05‐level test was conducted by calculating a 95% confidence interval for the difference in these two probabilities found using the method of Tango [21].

The probability of reporting itchiness (patient RISRAS grade 3: very much) was compared under the two treatment conditions using a two‐sided McNemar test.

### 2.9 Sample Size

Sample size was calculated for the primary null hypothesis. Based on the assumption that the absolute risk of MD might be 3% greater with gel than with film, we calculated that a sample size of 120 would give a power of 0.71 to reject the null hypothesis.

## 3 Results

### 3.1 Recruitment and Demographics

The trial recruited between June 2021 and April 2024. Because of department resources, not all eligible patients were offered participation in the trial and not all participating hospitals contributed for the full enrolment period (Table 1).

**TABLE 1:** Enrolment period and recruitment of the participating public hospitals.

Public hospital	Enrolment period	# Recruited	# Completed
Christchurch	July 2021–April 2024	65	57
Palmerston North	August 2021–January 2023	22	19
Wellington	July 2023–October 2023	6	4
**Total**		**93**	**80**

**FIGURE 1:**
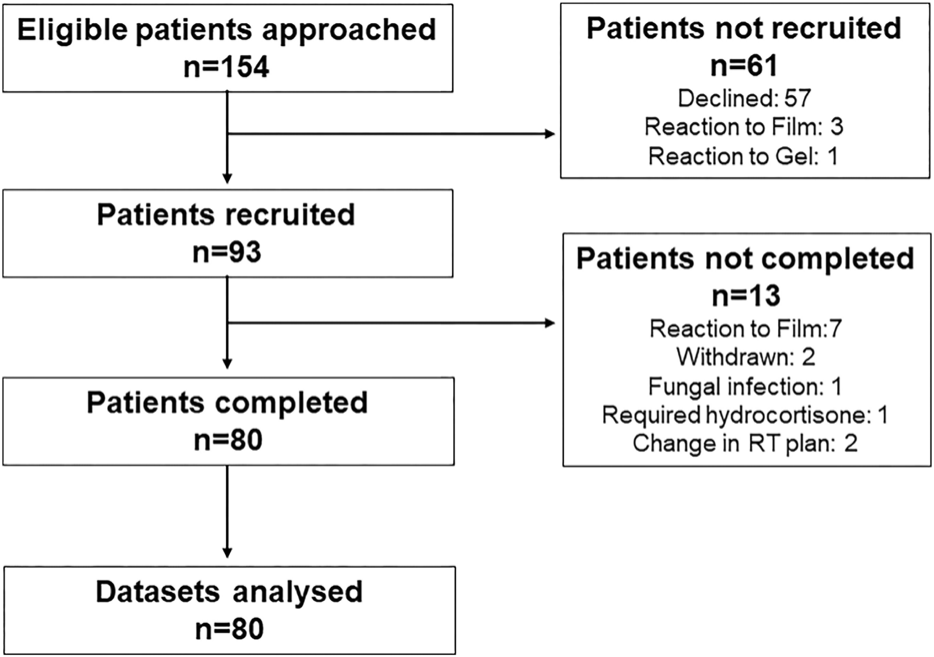
Consort diagram. Flow of participants through the trial.

Flow of patients through the trial is shown in the Consort diagram in Figure 1. A total of 57 patients declined participation, mainly because they were unable to commit to the follow‐up visits after completion of treatment. Three patients reacted to the Mepitel film test patch and one patient to StrataXRT. Out of 93 patients recruited, 80 completed the trial and their data sets were analysed. Seven patients pulled out of the trial because their skin reacted to Mepitel film. All participants were female with an average age of 59 years. The vast majority (81%) identified as NZ European, with 10% identifying as Māori, 8% as Asian and 1% as Pacific peoples. Patient‐ and treatment‐related factors are shown in Table 2. Randomisation to the lateral and medial sides of the chest wall was relatively well balanced with Mepitel film in 42 participants and StrataXRT in 38 participants applied to the lateral chest wall.

**TABLE 2:** Demographics: Patients, cancer, and treatment characteristics of the patient cohort.

Patient characteristics		*n* = 80
Gender	Female	80
Age in years	Average	59
	Range	(36–81)
Ethnicity	NZ European/European	65
	Maori	8
	Asian	6
	Pacific peoples	1
Fitzpatrick skin type	I	6
	II	37
	III	32
	IV	4
	V	1
Smoking	Current	5
	Past	31
	Never	44
Alcohol consumption	Never	32
	< 1 per week	25
	1–3 per week	15
	> 3 week	8
Sun exposure	Rarely	58
	Often	22
**Cancer and treatment characteristics**		
Cancer Grade	1	3
	2	36
	3	41
RT Fractionation	26Gy/5#	7
	40.05Gy/15#	70
	50Gy/25#	3
Technique	3D conformal	28
	IMRT/VMAT	52
Bolus (< 5 mm)	Yes	30
Chemotherapy	Yes	62
Hormone therapy	Yes	47

### 3.2 Treatment Effectiveness

#### 3.2.1 Moist Desquamation

**FIGURE 2:**
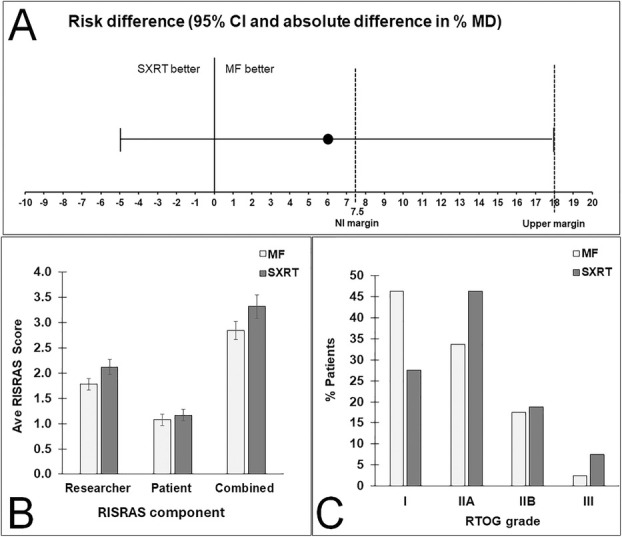
Comparisons of skin patches covered by Mepitel film and StrataXRT. (A) Confidence interval for the difference in probability of moist desquamation. (B) Average RISRAS scores. (C) Relative frequencies of RTOG grades. MF, Mepitel film; SXRT, StrataXRT.

Of the 80 patients, 51 did not develop MD under either intervention, 13 had MD under StrataXRT but not under Mepitel film, 8 had MD under Mepitel film but not under StrataXRT, and 8 had MD under both interventions. The risk of MD was 6% higher under StrataXRT (21/80; 26%) than under Mepitel film (16/80; 20%). The upper limit of the two‐sided 95% interval (18%) sits well above the non‐inferiority margin of 7.5% (Figure 2A), meaning we cannot conclude that StrataXRT is non‐inferior to Mepitel film at the 7.5% margin (*p* = 0.413) (Table 3).

**TABLE 3:** Results of statistical analysis for skin reaction severity.

Measure	*p*	Point estimate (95% CI)
Moist desquamation^a^	0.413	6% (−5, 18)
RISRAS—Researcher^b^	0.022	
RISRAS—Patient^b^	0.815	
RISRAS—Combined^b^	0.204	
RTOG^c^	0.011	21% (5,36)

^a^ Estimate is of probability of MD under gel minus probability of MD under film. P‐value is from hypothesis test that probability of MD under gel is greater than or equal to probability of MD under film plus 0.075.

^b^ Non‐parametric test: no parameters estimated. *p* is from the hypothesis test that the distributions of scores under gel and film are the same.

^c^ Estimate is the probability that grade under gel exceeds the grade under film minus the probability that the grade under film exceeds the grade under gel. *p* value is from the hypothesis test that the probability that the grade under gel exceeds the grade under film is equal to the probability that the grade under film exceeds the grade under gel.

#### 3.2.2 RISRAS Scores and RTOG Grades

Average researcher, patient and combined RISRAS scores for both interventions are shown in Figure 2B. Scores were lower for Mepitel film for all three RISRAS components, but this was statistically significant only for the researcher component (*p* = 0.022) (Figure 2B, Table 3).

The percentages of patients that scored RTOG grades I, IIA, IIB and III for skin underneath Mepitel film and StrataXRT are shown in Figure 2C. Of the 80 patients, 31 had a higher RTOG grade under StrataXRT than under Mepitel film, 14 had a higher RTOG grade under Mepitel film than under StrataXRT, and 35 had the same grade under StrataXRT and Mepitel film. This shows that patients were more likely to have a higher RTOG grade under StrataXRT (*p* = 0.011) (Figure 2C, Table 3).

### 3.3 Tolerability and Satisfaction

Previous trials have mentioned the issue of itchiness underneath Mepitel film. Seven participants pulled out of the trial because of skin reactions to Mepitel film. Out of the remaining 80 participants who completed the trial, nine participants scored only Mepitel film‐covered skin as very itchy (patient RISRAS itchiness: 3), four participants scored only StrataXRT‐covered skin as very itchy, and one participant scored skin underneath both interventions as very itchy. The *p*‐value in the corresponding two‐sided McNemar test of the hypothesis of no difference in skin itch is *p* = 0.267.

A total of 63 patients (79%) returned the exit questionnaire. All responders reported that participating in the trial had been a positive experience for them. Good communication, helpful and supportive staff, and weekly check‐ups were noted by 27 responders, whilst 16 responders wanted to help future cancer patients by finding better ways to avoid severe skin reactions. With respect to preference, 20 responders preferred Mepitel film, 27 responders preferred StrataXRT and 16 responders did not have a preference. Positive and negative features of both interventions are displayed in Table 4. The main positives were not having to worry about applying Mepitel film and the ease of applying StrataXRT themselves. Peeling at the edges and the need to replace Mepitel film were the main negatives, as were the twice‐daily application and waiting for the StrataXRT to dry.

**TABLE 4:** Responders' comments on the use of Mepitel film and StrataXRT.

	# (%) responders
**Mepitel film**	
*Positive*	
I did not have to worry or do anything myself	31 (50)
Felt more protective/less discomfort	11 (18)
*Negative*	
Rolling at the edges, did not stick in skin folds	28 (45)
Not able to shower/wash properly	7 (11)
Itchy	4 (6)
Visible under certain clothing	3 (5)
**StrataXRT**	
*Positive*	
Ease of application	23 (37)
Invisible	2 (3)
Could shower normally	2 (3)
Soothing when XRT put in fridge	2 (3)
Better in skin folds	3 (5)
*Negative*	
Time it takes to apply twice daily and wait to dry	28 (45)
Remembering to apply the XRT	9 (15)
Worried the amount of gel put on was not enough	4 (6)
Stained clothing/sheets	3 (5)
Itchy	3 (5)

### 3.4 Costs

We compared the costs associated with both interventions, including the time spent by radiation therapists applying and reapplying Mepitel film. We note here that only half of the chest wall was covered with either Mepitel film or StrataXRT. We used 240 sheets of Mepitel film ($9.974 per sheet; https://www.capesmedical.co.nz/shop/Woundcare/Dressing/Film/Mepitel) and 115 large tubes ($229.95 per tube) and 5 small tubes ($119.45 per tube) of StrataXRT (https://nz.stratpharma‐shop.com/product‐category/StrataXRT). Radiation therapists spent on average 20 min applying one sheet of Mepitel film, adding another hour (3 sheets) per patient at NZ $45 to the costs, with a total average cost per patient of NZ $75 for Mepitel film and NZ $338 for StrataXRT.

## 4 Discussion

This RCT compared the protective effects on irradiated skin by two silicone‐based interventions: a film (Mepitel film) applied by radiation therapists and a gel that forms a film upon drying (StrataXRT) applied by patients themselves. We observed that the percentage of trial participants with moist desquamation under StrataXRT was 6% higher (absolute difference) than under Mepitel film. The upper limit of the 95% CI of 18% was greater than the pre‐specified non‐inferiority margin of 7.5%, so we cannot conclude that StrataXRT is non‐inferior to Mepitel film with respect to MD.

To the authors' knowledge, only one very recently published RCT directly compares the protective effects on irradiated skin of Mepitel film with StrataXRT, using a similar randomised methodology to the current trial [22]. Lee et al. report that StrataXRT was inferior to Mepitel Film with respect to time‐weighted average (TWA) grade of skin reaction severity (non‐inferiority threshold 25%) but non‐inferior with respect to worst skin reaction grade (non‐inferiority threshold 50%) in 40 postmastectomy patients. In contrast to our trial, Lee et al. showed that more patients developed MD under Mepitel film compared to StrataXRT (20% and 12.5% respectively). Similar to the current trial, patient preferences were evenly divided in the Lee et al. trial [22].

Researcher RISRAS scores and RTOG grades were significantly lower for Mepitel film, but StrataXRT was better tolerated. Three patients, who developed a rash underneath a Mepitel film test patch, were not recruited, and a further seven patients withdrew because of severe itching, erythema and heat underneath Mepitel film. In addition, 10 out of 80 patients scored skin underneath Mepitel film as “very itchy” (Patient RISRAS). The trial by Lee et al. reported one participant with itching under StrataXRT and three participants under Mepitel film, which resulted in the removal of one of these from the trial. Intolerance to Mepitel film was also responsible for six out of 251 patients leaving the trial by Behroozian et al. [13] and 14 out of 101 patients by Moller et al. [12]. Three patients in the trial by Chao developed itching underneath Mepitel film, with one patient pulling out of the trial early [22]. Poor tolerance of Mepitel film, with itchiness, tightness and discoloration in 13 out of 28 patients, resulted in early closure of a head and neck cancer trial by Rades et al. [16]. Three other trials also mentioned discoloration and itchiness under Mepitel film in three out of 78 breast cancer patients [4], eight out of 33 head and neck cancer patients [6], and 28 out of 44 head and neck cancer patients [7], but these patients completed their trials. It is obvious from this and previous trials that some patients do not tolerate Mepitel film on their skin. Poor skin adherence, particularly in the axilla, and curling up at the edges, as well as interference with showering and bathing, were all mentioned as negative aspects of Mepitel film in previous trials [4, 6, 7, 12, 13, 16] as well as in the current trial.

A recent comprehensive review by Kuszaj et al. [15] on costs associated with using Mepitel film mentions that the number of sheets per patient depends mainly on the type of surgery (mastectomy or lumpectomy) and breast size. We applied both interventions to half of the chest wall and found Mepitel film much cheaper to apply, including radiation therapist time. However, time spent on application is not just a financial resource but also impacts the flow of patients through the department. The latter is the most likely reason that many centres in New Zealand are now using StrataXRT. The possibility of training the partner/relatives of patients to apply Mepitel film rather than healthcare professionals was discussed by Rajeswaran et al. [23], who analysed the perceptions of Canadian health care professionals on the use of Mepitel film for radiation dermatitis in breast cancer patients. A change in breast shape by incorrect positioning of Mepitel film is not an issue for mastectomy patients, and having partners/relatives apply Mepitel film may increase the uptake of the film in more centres.

### 4.1 Strengths and Limitations

A major strength of this trial is its intra‐patient‐controlled design, with all patients experiencing both interventions at the same time, removing potentially confounding patient‐ and treatment‐related confounders in inter‐patient‐controlled trials. The main limitation is the lack of blinding, which is inherent in skin trials that use two visually different products. Another limitation is that our sample size was smaller than originally planned (*n* = 80 rather than *n* = 120) due to resource constraints and recruitment challenges during and immediately after the COVID‐19 pandemic.

**In summary**, in this study StrataXRT did not meet the non‐inferiority threshold for moist desquamation rates in 80 mastectomy patients. In addition, visual skin reactions were significantly less severe under Mepitel film. However, StrataXRT was better tolerated, and while Mepitel film was cheaper to apply, poor adherence of the film in skin folds remains an important issue. This trial recommends both interventions for the management of skin reactions in breast cancer patients, with application depending on patient‐specific factors such as mastectomy vs. lumpectomy, departmental resources and patient wishes.

## Ethics Statement

Ethical approval was obtained from the Otago Ethics Committee in November 2020 (H20/130) and from the Northern B Health and Disability Ethics Committee in April 2021 (21/NTB/78).

## Consent

All participants gave written informed consent before the start of radiation therapy treatment.

## Conflicts of Interest

The authors declare no conflicts of interest.

## Data Availability

De‐identified data will be made available upon reasonable request.
